# Factors Influencing Outcome After Frontal Beak Reduction—Does the Surgical Tool Matter?

**DOI:** 10.3390/jcm14238377

**Published:** 2025-11-26

**Authors:** Łukasz Skrzypiec, Kornel Szczygielski, Dariusz Jurkiewicz, Marta Aleksandra Kwiatkowska

**Affiliations:** Department of Otolaryngology and Laryngological Oncology with Clinical Department of Cranio-Maxillofacial Surgery, Military Institute of Medicine—National Research Institute, 128 Szaserów Street, 04-141 Warsaw, Poland

**Keywords:** frontal surgery, quality of life, piezo surgery, frontal recess stenosis, endoscopic sinus surgery, chronic rhinosinusitis

## Abstract

**Introduction**: Chronic rhinosinusitis (CRS) with frontal sinus outflow tract (FSOT) obstruction frequently requires frontal beak reduction during endoscopic sinus surgery (ESS). While technical approaches such as rotary drilling or piezoelectric osteotomy may differ in precision and tissue preservation, it is unclear whether surgical instrument choice or patient-specific clinical characteristics influence postoperative quality of life (QoL). **Methods**: In this prospective cohort study, 49 adult CRS patients undergoing ESS with frontal beak reduction were enrolled (28 males, mean age 50 ± 15 years). Osteotomy was performed using either a conventional drill (n = 25) or piezoelectric knife (n = 24). Baseline clinical data included presence of nasal polyps, asthma, and radiologic severity (Lund–Mackay and Zinreich CT scores). Outcomes included patient-reported symptoms with the 22-item Sino-Nasal Outcome Test (SNOT-22) and visual analogue scale (VAS), and endoscopic Lund–Kennedy scores, recorded preoperatively and at 1, 4, and 24 weeks postoperatively. **Results**: Both groups demonstrated significant postoperative improvement in SNOT-22 and VAS scores. No significant correlation was observed between SNOT-22 changes and Lund–Kennedy scores in either tool group. Presence of nasal polyps was associated with higher pre- and postoperative Zinreich and Lund–Mackay scores (*p* < 0.05). Asthma was linked to higher early postoperative symptom burden (nasal blockage, clear discharge; *p* < 0.05). Tool choice did not significantly influence QoL recovery or modify the effect of clinical characteristics on outcomes. **Conclusions**: Postoperative QoL improvement was driven primarily by baseline disease phenotype—particularly nasal polyposis and asthma—while the choice of osteotomy instrument did not significantly influence recovery trajectories. The Zinreich score provided additional phenotypic stratification in CRS with FSOT obstruction.

## 1. Introduction

Chronic rhinosinusitis (CRS) involving the frontal sinus presents a distinct surgical challenge due to the complex anatomy and narrow frontal sinus outflow tract (FSOT). In many cases, a prominent nasofrontal beak contributes to FSOT obstruction, necessitating its removal during endoscopic sinus surgery (ESS) to restore ventilation and drainage. Successful treatment aims not only to improve objective endoscopic and radiologic parameters but also to enhance patient-reported quality of life (QoL)**,** which has become a key benchmark in contemporary CRS outcomes research [1,2].

CRS represents a heterogeneous disease with distinct inflammatory endotypes that markedly influence clinical severity, polyp formation, and postoperative outcomes [3]. Increasing evidence supports the “united airway” concept, whereby upper airway inflammation parallels and often depends on the control of lower airway disease, particularly asthma. Endotypic characterization—such as type 2–dominant inflammation associated with nasal polyposis and asthma—has been shown to correlate with greater radiologic burden and more aggressive frontal recess disease [4]. Considering these systemic factors is therefore essential when evaluating anatomical contributors to FSOT obstruction and interpreting postoperative outcomes.

Multiple factors may influence postoperative QoL improvement after frontal sinus surgery. Clinical characteristics such as nasal polyposis and asthma have been linked to greater disease severity, higher recurrence rates, and less favourable QoL gains following ESS [3,5,6]. Radiologic severity, commonly assessed with the Lund–Mackay system, has been shown to correlate variably with symptom burden, and more refined grading methods such as the Zinreich scale allow targeted assessment of FSOT stenosis [7,8,9,10]. However, the predictive value of these factors specifically after frontal beak reduction has not been well established.

In addition, the choice of surgical tool for frontal beak osteotomy—whether a conventional high-speed drill or a piezoelectric knife—may influence intraoperative precision, mucosal preservation, and healing dynamics. Piezoelectric devices have demonstrated reduced soft-tissue trauma and improved bone healing in maxillofacial and dental surgery [11,12,13], but comparative data in frontal sinus surgery are sparse. Whether such technical differences translate into meaningful QoL benefits or interact with patient-specific factors remains unknown.

To our knowledge, no previous study has simultaneously examined the impact of baseline clinical characteristics and surgical instrument choice on postoperative QoL following frontal beak reduction. As this procedure, when anatomically indicated, is a key step to achieving a ≥4–5 mm durable frontal outflow tract, inadequate technique increases the likelihood of recurrent obstruction, persistent symptoms, and revision surgery.

To date, no study has systematically examined the interplay between baseline clinical characteristics and the choice of osteotomy instrument in the specific context of frontal beak reduction. While previous work has addressed piezoelectric osteotomy in sinonasal surgery, including our own pilot study suggesting improved soft-tissue preservation [14] prospective validation of these findings and their relevance to postoperative quality-of-life recovery has been lacking. Furthermore, the integration of the Zinreich CT scoring system offers an anatomically focused assessment of FSOT stenosis that complements the global Lund–Mackay score.

Presented prospective analysis aims to determine whether nasal polyposis, asthma, radiologic severity, and the type of osteotomy instrument (drill vs. piezoelectric knife) influence QoL recovery in patients undergoing ESS for CRS with FSOT obstruction.

## 2. Material and Methods

Research was designed as a prospective, single-centre study conducted at the Military Institute of Medicine—National Research Institute in Warsaw, Poland. The study protocol was approved by the Institutional Research Ethics Committee (protocol number 75/WIM/2017, approval date: 15 November 2017). Written informed consent was obtained from all participants prior to enrollment.

Consecutive adult patients diagnosed with chronic rhinosinusitis according to the European Position Paper on Rhinosinusitis and Nasal Polyps (EPOS) guidelines were enrolled if there was a lack of improvement following a minimum of 12 weeks of medical treatment with topical corticosteroids, with or without antibiotics [3].

Exclusion criteria were concomitant septoplasty, history of previous sinonasal surgery or facial trauma involving the sinonasal complex, Samter’s triad (aspirin sensitivity, asthma, nasal polyposis), sinonasal neoplastic or obstructive lesions, known ciliary dysfunction, cystic fibrosis, osteoneogenesis, or Paget’s disease, bleeding disorders or anticoagulant therapy, severe systemic or neuropsychiatric disorders.

Although asthma was analyzed as a clinical variable, all patients with aspirin-exacerbated respiratory disease (AERD/Samter’s triad) were excluded. Therefore, the asthma subgroup reported in the Results corresponds exclusively to non-AERD asthma.

### 2.1. Preoperative Evaluation

All patients underwent comprehensive otolaryngological assessment, including nasal endoscopy and high-resolution computed tomography (CT) of the paranasal sinuses analyzed in axial, coronal, and sagittal planes. The degree of frontal sinus outflow tract stenosis was measured, and the presence of a prominent nasofrontal beak was assessed.

Radiologic staging was performed using both the Lund–Mackay system and the Zinreich CT scoring system. Endoscopic findings were graded according to the Lund–Kennedy scoring system [15].

The Zinreich scale is a computed tomography-based radiological grading system designed to quantify the severity of frontal sinus outflow tract obstruction, particularly in the context of frontal sinusitis. The scale assesses the degree of narrowing in the frontal recess and is graded from 0 (no obstruction) to 5 (complete obstruction with associated opacification), with higher scores indicating more severe stenosis.

In this study, Zinreich grading was performed preoperatively and repeated 24 weeks after surgery to assess changes in frontal sinus drainage pathway patency.

Comorbidities were systematically recorded, including the presence of asthma, nasal polyposis, allergic rhinitis, and other chronic respiratory conditions. Asthma was defined according to current clinical guidelines [16] and classified as non-aspirin-exacerbated respiratory disease (AERD), since patients with Samter’s triad were excluded. For the purposes of subgroup analysis, patients were stratified based on the presence or absence of asthma and nasal polyposis at baseline.

The distribution of comorbidities was comparable between the drill and piezoelectric groups. No significant differences were observed in the prevalence of asthma, nasal polyposis, or allergic rhinitis between the two surgical cohorts, indicating that both groups were matched with respect to major clinical characteristics.

### 2.2. Surgical Procedure

Surgical procedures included endoscopic ethmoidectomy, maxillary antrostomy, sphenoidectomy, and frontal sinus outflow tract exposure. The osteotomy of the prominent nasofrontal beak was performed using one of two techniques: in the “drill group” conventional rotary instruments (Straightshot™ M5 microdebrider, Medtronic^®^, Jacksonville, FL, USA) with single-use burs (30K reverse tapered diamond bur, 4.0 mm, 70°) were applied, whereas in the “piezo” group the ultrasonic osteotomy system (Piezotome SOLO M+^®^, Satalec, Merignac, France) with single-use application tips (long ultra-sharp saw BS1 XXL and long scalpel BS6 XXL) was used.

### 2.3. Outcome Measures

The Lund–Kennedy endoscopic scoring system was applied preoperatively and at 1, 4, and 24 weeks postoperatively. It evaluates polyps, discharge, edema, scarring, and crusting (0–2 points per item, per side; total 0–10 points), with higher scores indicating more severe disease.

The Lund–Mackay scoring system was applied to CT scans preoperatively and at 24 weeks postoperatively, assigning scores from 0 (no opacification) to 2 (complete opacification) for each paranasal sinus and the ostiomeatal complex, yielding a total of 0–24 points.

The Zinreich scale was used for frontal sinus outflow tract assessment as well, as described above.

### 2.4. Symptom and Quality-of-Life Assessment

The 22-item Sino-Nasal Outcome Test (SNOT-22) was administered preoperatively and at 1, 4, and 24 weeks postoperatively.

With Visual Analogue Scale (VAS) patients rated the severity of seven core rhinosinusitis symptoms separately as follows: (a) facial/ocular pressure, (b) headache/facial pain, (c) nasal blockage, (d) clear nasal discharge, (e) purulent nasal discharge (yellow/green/brown), (f) nasal itching, g) epistaxis. Each symptom was scored from 0 (no discomfort) to 10 (maximum discomfort) using a 10 cm VAS [17]. Evaluations were performed immediately postoperatively (day 0) and at 7 days postoperatively.

### 2.5. Statistical Analysis

Statistical analysis was performed using Statistica 13.0. The conformity of the distribution of quantitative variables to a normal distribution was checked using the Shapiro–Wilk test. Quantitative data were presented using basic descriptive statistics (mean, SD, median, range), and qualitative data were presented in the form of percentage distribution of results. To verify the hypotheses set for the study, the U-Mann–Whitney test was used. To test the direction and strength of correlation between variables, Pearson’s correlation coefficient or Spearman’s rank coefficient were determined, respectively. The level of statistical significance was set at 0.05.

## 3. Results

A total of 49 patients (28 males [57.1%] and 21 females [42.9%]) were included in the study. The mean age of the cohort was 50 ± 15 years, with a median of 48 years (range: 26–73 years). In 25 patients (49%), prominent nasofrontal beak removal was performed using a drill, while in 24 patients (51%) a piezoelectric knife was used for osteotomy.

Patients’ symptoms were assessed using a visual analogue scale (VAS) for seven major rhinosinusitis symptoms, evaluated separately for each nasal cavity: (a) facial and ocular pressure, (b) headache or facial pain, (c) nasal blockage, (d) clear nasal discharge, (e) purulent (yellow, green, or brown) nasal discharge, (f) nasal itching, (g) epistaxis.

For the entire cohort, no statistically significant correlation was observed between VAS scores on the day of surgery and Lund–Kennedy scores, except for a significant negative low correlation between VAS item (c)—nasal blockage—and the total Lund–Kennedy score.

In the subgroup of patients treated with the piezoelectric knife, no statistically significant correlations were found between VAS scores (both on postoperative day 0 and at 1 week) and Lund–Kennedy scores.

In the drill subgroup, no significant correlations were found between VAS scores on postoperative day 0 and Lund–Kennedy scores. However, at 1 week postoperatively, a statistically significant negative moderate correlation was observed between VAS item (c)—nasal blockage—and the Lund–Kennedy score.

When considering the entire cohort and the piezoelectric knife subgroup, no statistically significant associations were observed between VAS scores (day 0 and 1 week postoperatively) and Lund–Kennedy scores at 1 week. In contrast, in the drill subgroup, a statistically significant moderate positive correlation was found only between the Lund–Kennedy score at 1 week and VAS item (f)—nasal itching. The exact results are given in Table 1.

When patients were stratified according to the presence of nasal polyps, a statistically significant difference between groups was observed for VAS items (b)—headache/facial pain—and (e)—purulent nasal discharge—on postoperative day 0, and for item (c)—nasal blockage—at 1 week postoperatively. At 1 week, the group without polyps demonstrated a significantly lower score for VAS item (e) and a significantly higher score for VAS item (c) compared with the group with polyps.

It should be noted that patients with Samter’s triad were excluded; thus, asthma-related comparisons reflect only non-AERD asthma.

When patients were stratified according to the presence of asthma, statistically significant differences between groups were observed for VAS items (b)—headache/facial pain, (c)—nasal blockage, (d)—clear nasal discharge, and (g)—epistaxis on postoperative day 0, as well as for items (c) and (d) at 1 week postoperatively. The exact results are given in Table 2.

**Figure 1:**
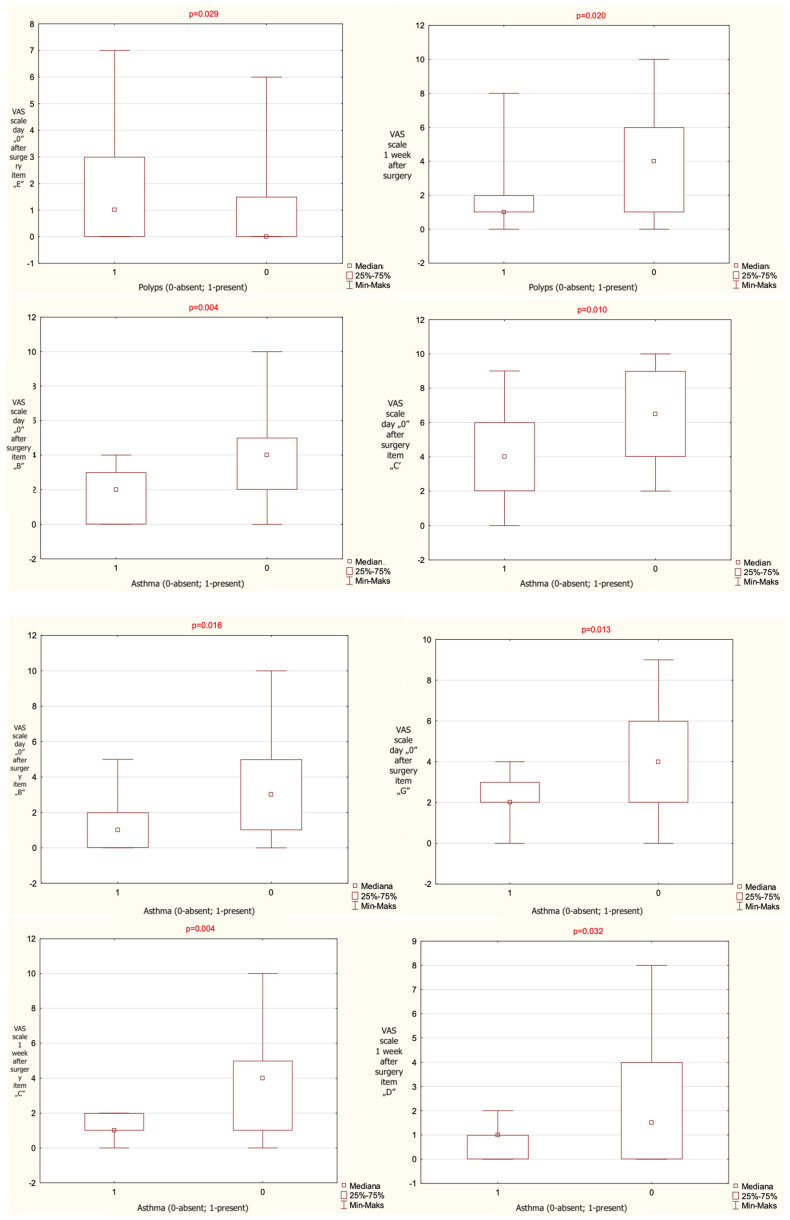
The results given as mean value, range and median, observed for the entire cohort regarding VAS items significant statistically and presence or absence of polyps on nasal endoscopy and asthma, noted on the day of surgery and 1 week after surgery (*p*-level of statistical significance). VAS—Visual Analogue Scale.

The results that have reached statistical significance are presented on Figure 1.

**Figure 2:**
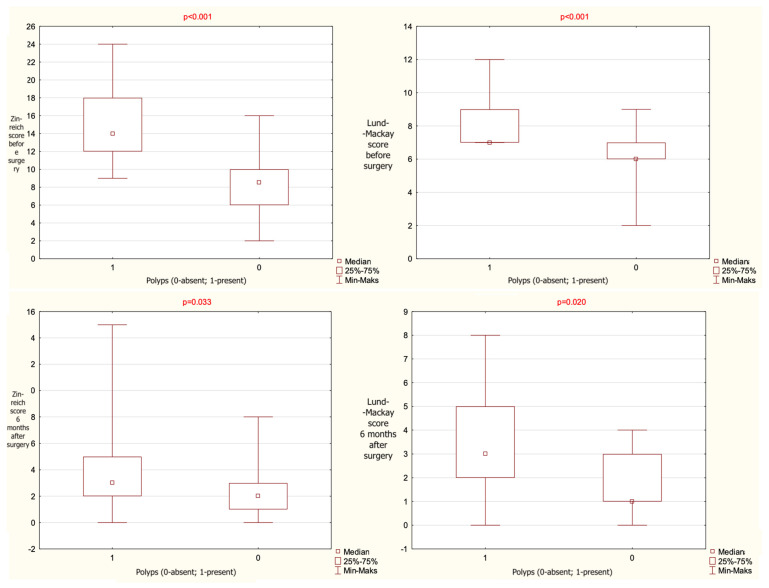
The results given as mean value, range and median, observed for the entire cohort regarding the presence or absence of polyps and the total score of computed tomography (CT) analysis with the use of Zinreich and Lund–Mackay scales (*p*-level of statistical significance).

A statistically significant association was found between Zinreich CT scores and the presence of nasal polyps. Patients without polyps had significantly lower Zinreich scores both preoperatively and 24 weeks postoperatively. A similar relationship was observed for Lund–Mackay scores. The statistical data are given in Table 3 and presented graphically on Figure 2.

No association was found between the reduction in sinonasal symptoms assessed using the SNOT-22 scale and the endoscopic appearance of the paranasal sinuses evaluated using the Lund–Kennedy scale. The exact data are given in Table 4.

## 4. Discussion

Presented work focus on assessment of frontal beak reduction, an anatomical step that is critical yet often underreported in outcome studies of frontal sinus surgery. By combining patient-reported outcomes, endoscopic evaluation, and anatomically targeted radiologic scoring, our study extends the observations of earlier pilot data [14] and provides prospective validation in a larger, clinically heterogeneous cohort.

Available prospective and cohort data show significant postoperative improvement in SNOT-22 after frontal surgery. Patients undergoing more advanced procedures (like Draf II/III) typically start with worse baseline scores but realize large postoperative gains, approaching the QoL of less-severe cohorts [18,19]. Early and mid-term failures of frontal sinusotomy correlate with severe preoperative disease and adverse anatomy of the frontal outflow tract; inadequate enlargement (including insufficient beak reduction) predisposes to recurrent obstruction and revision surgery [20].

Presented research revealed that health-related quality of life (HRQoL)**,** assessed with the SNOT-22 questionnaire, improved substantially in both surgical groups over the postoperative follow-up period as well. However, no statistically significant correlation was found between changes in SNOT-22 scores and endoscopic findings as graded by the Lund–Kennedy scale. This aligns with evidence that postoperative symptom burden does not necessarily parallel early endoscopic changes [21,22,23].

When analyzing clinical characteristics, the presence of nasal polyps was associated with higher preoperative radiologic disease burden, as reflected by both Zinreich and Lund–Mackay scores. Patients without polyps had significantly lower radiologic scores preoperatively and maintained this advantage at 24 weeks postoperatively. This correspond with literature showing that nasal polyposis is linked to more extensive mucosal disease and a higher rate of frontal recess involvement [3,24]. Importantly, obtained data confirm that polyp status should be considered when interpreting radiologic severity and predicting postoperative outcomes. The association between comorbidities and radiologic severity in our cohort further supports the potential of the Zinreich score as a phenotypic marker in CRS with FSOT obstruction. Patients with asthma and nasal polyposis—conditions typically aligned with type 2 inflammatory endotypes—demonstrated higher Zinreich scores both pre- and postoperatively. These findings reinforce that comorbid respiratory disease and its level of control contribute to the anatomical and mucosal changes underlying frontal recess obstruction. By capturing the degree of frontal recess stenosis with greater specificity than global CT staging systems, the Zinreich scale may therefore serve as a valuable adjunct for phenotypic stratification in CRS.

Regarding asthma comorbidity, patients with asthma reported significantly worse symptom scores for nasal blockage, clear nasal discharge, and other sinonasal complaints in the early postoperative period. This is in line with previous studies demonstrating that asthma is a marker of more severe and persistent CRS symptoms and may predict slower postoperative recovery [6,25]. The shared inflammatory mechanisms of upper and lower airway disease likely contribute to this pattern, as proposed in the “united airway” concept [26].

From a radiologic perspective, the integration of Zinreich grading allowed to quantify frontal recess stenosis severity more specifically beyond the global Lund–Mackay score. The observed correlation between polyp status and Zinreich scores both pre- and postoperatively provides novel insight, suggesting that the Zinreich system could serve as a radiologic biomarker for disease phenotype and surgical complexity. By incorporating this radiologic tool, the present study provides a more refined evaluation of frontal recess anatomy and its impact on clinical outcomes.

When patients were stratified by the instrument used for frontal beak reduction, no statistically significant correlations were found between SNOT-22 scores and Lund–Kennedy endoscopic findings at any postoperative time point in either the drill or the piezoelectric knife groups. This suggests that the choice of osteotomy tool did not influence the relationship between patient-reported QoL and objective endoscopic appearance [18,27].

Despite these findings, qualitative intraoperative observations and existing literature suggest certain practical advantages of ultrasonic instruments. Piezoelectric systems have been associated with reduced soft-tissue trauma, decreased thermal injury risk, and enhanced precision of bone removal in maxillofacial and sinonasal procedures [11,12]. In previously published pilot study [14], mucosal preservation appeared subjectively improved with piezoelectric osteotomy; however, quantitative measures of mucosal trauma or bleeding were not collected in the current cohort, and this observation should therefore be interpreted cautiously. It is also important to acknowledge practical considerations such as the steeper learning curve, increased operative time reported in some studies, and higher equipment cost, all of which may influence the selection of surgical tools despite comparable clinical outcomes.

For radiologic severity, as assessed by the Zinreich and Lund–Mackay scores, both groups demonstrated postoperative improvement, but no significant tool-related differences in correlation with polyp status, asthma, or other clinical characteristics were observed. Polyp presence remained associated with higher radiologic scores pre- and postoperatively in both groups, indicating that disease phenotype rather than instrument choice drives radiologic burden [4,5].

Similarly, VAS symptom analysis revealed that significant correlations between specific symptoms and endoscopic scores were more a function of patient comorbidities (e.g., asthma, nasal polyps) than the surgical device employed. The moderate correlation between nasal blockage (VAS item c) and Lund–Kennedy scores at 1 week was present only in the drill group, while no such correlation emerged in the piezoelectric group—suggesting that subtle postoperative mucosal differences might exist, but without translating into measurable differences in QoL scores. Nowadays, the clinical importance of preventing restenosis is further supported by adjunctive therapies that specifically target the frontal ostium.

Steroid-releasing implants placed in the frontal recess reduce inflammation and restenosis—an intervention whose benefit highlights how restenosis of a suboptimally widened ostium (e.g., after inadequate frontal beak reduction) is a key driver of subpar outcomes [28]. Nevertheless, if steroid-releasing implants are not available in healthcare system provided, effective bony reduction in frontal recess is closely tied to patients’ QoL gains [14].

## 5. Limitations

The primary limitation of this study is the modest sample size, which reduces the statistical power for detecting subtle intergroup differences, particularly regarding potential interactions between clinical characteristics and the choice of osteotomy instrument. As a result, non-significant findings—especially those related to tool comparison—should be interpreted with caution due to the increased risk of type II error. Larger multicenter cohorts will be essential to validate the observed trends and provide more robust estimates of effect size.

## 6. Conclusions

Taken together, these findings indicate that the type of osteotomy instrument does not substantially alter QoL recovery trajectories or the impact of baseline clinical characteristics on postoperative outcomes. Instead, factors such as nasal polyposis and asthma status remain the dominant drivers of both subjective and objective measures.

## Figures and Tables

**Table 1:** Spearman’s rank test results of endoscopic Lund–Kennedy results on the day of surgery and one week after surgery and the visual analogue scale (VAS) sinonasal items. Results given for the entire cohort and divided into surgical subgroups according to the surgical tool used for the frontal beak reduction (N-number, R- correlation strength [effect size], t(N-2)—the test statistic for significance testing, *p*-value—level of statistical significance). VAS items: (a) facial and ocular pressure, (b) headache or facial pain, (c) nasal blockage, (d) clear nasal discharge, (e) purulent (yellow, green, or brown) nasal discharge, (f) nasal itching, (g) epistaxis.

Endoscopic Lund–Kennedy Results at the Day of Surgery	Spearman’s Rank Test Results for the Entire Cohort				Spearman’s Rank Test Results for the Piezoelectric Knife				Spearman’s Rank Test Results for Drill			
VAS-scale item	N	R	t(N-2)	*p*-value	N	R	t(N-2)	*p*-value	N	R	t(N-2)	*p*-value
a	49	−0.152	−0.723	0.477	24	−0.041	−0.281	0.780	25	0.080	0.383	0.705
b	49	−0.365	−1.838	0.080	24	−0.201	−1.405	0.167	25	−0.018	−0.085	0.933
c	49	−0.216	−1.039	0.310	24	−0.244	−1.727	0.091	25	−0.322	−1.630	0.117
d	49	−0.108	−0.509	0.616	24	−0.164	−1.141	0.260	25	−0.160	−0.780	0.444
e	49	0.145	0.686	0.500	24	0.117	0.809	0.422	25	0.166	0.808	0.427
f	49	−0.348	−1.740	0.096	24	−0.145	−1.006	0.320	25	0.059	0.282	0.780
g	49	−0.102	−0.483	0.634	24	−0.195	−1.362	0.180	25	−0.354	−1.813	0.083
Endoscopic Lund–Kennedy results 7 days after surgery	Spearman’s rang test results for the entire cohort				Spearman’s rang test results for the piezoelectric knife				Spearman’s rang test results for drill			
VAS-scale item	49	−0.062	−0.290	0.775	24	−0.164	−1.142	0.259	25	−0.281	−1.402	0.174
b	49	−0.395	−2.017	0.056	24	−0.277	−1.974	0.054	25	−0.163	−0.790	0.437
c	49	−0.240	−1.159	0.259	24	−0.391	−2.909	0.006	25	−0.540	−3.076	0.005
d	49	0.023	0.108	0.915	24	−0.104	−0.718	0.476	25	−0.218	−1.072	0.295
e	49	−0.014	−0.066	0.948	24	−0.121	−0.838	0.406	25	−0.243	−1.200	0.242
f	49	−0.241	−1.165	0.257	24	−0.142	−0.982	0.331	25	−0.070	−0.334	0.741
g	49	0.088	0.416	0.682	24	−0.014	−0.098	0.923	25	−0.220	−1.083	0.290

**Table 2:** U-Mann–Whitney test results for VAS items and presence or absence of polyps on nasal endoscopy and asthma presented on the day of surgery and 1 week after surgery. VAS items: (a) facial and ocular pressure, (b) headache or facial pain, (c) nasal blockage, (d) clear nasal discharge, (e) purulent (yellow, green, or brown) nasal discharge, (f) nasal itching, (g) epistaxis; U- Calculated based on the sum of ranks of the two groups being compared, Z- The standardized value of the U statistic, *p*-level of statistical significance. VAS—Visual Analogue Scale.

Analyzed Variable	U-Mann–Whitney Test for VAS and Polyps							U-Mann–Whitney Test for VAS and Asthma						
	Sum.Rang Polyps	Sum.Rang No Polyps	U	Z	N Polyps	N No Polyps	*p*-Value	Sum.Rang Asthma	Sum.Rang NO Asthma	U	Z	N Asthma	N No Asthma	*p*-Value
Day “0” after surgery														
a	688	538	253	−0.753	29	20	0.449	287	939	167	−1.909	15	34	0.054
b	617	609	182	−2.197	29	20	0.026	244	982	124	−2.842	15	34	0.004
c	649	576	214	−1.536	29	20	0.125	257	968	137	−2.549	15	34	0.010
d	687	538	252	−0.763	29	20	0.449	266	960	146	−2.365	15	34	0.016
e	832	394	184	2.156	29	20	0.029	409	816	221	0.727	15	34	0.472
f	751	475	265	0.509	29	20	0.607	335	891	215	−0.868	15	34	0.384
g	631	594	196	−1.902	29	20	0.057	261	964	141	−2.462	15	34	0.013
1 week after surgery														
a	689	537	254	−0.732	29	20	0.461	331	895	211	−0.954	15	34	0.338
b	668	558	233	−1.159	29	20	0.245	285	940	165	−1.942	15	34	0.051
c	611	614	176	−2.309	29	20	0.020	246	980	126	−2.798	15	34	0.004
d	688	538	253	−0.753	29	20	0.449	276	949	156	−2.137	15	34	0.032
e	706	519	271	−0.376	29	20	0.709	325	900	205	−1.074	15	34	0.286
f	736	490	280	0.203	29	20	0.832	307	919	187	−1.475	15	34	0.139
g	671	554	236	−1.088	29	20	0.279	305	921	185	−1.519	15	34	0.127

**Table 3:** The results of U-Mann–Whitney test observed for the entire cohort regarding the presence or absence of polyps and the total score of computed tomography (CT) analysis with the use of Zinreich and Lund–Mackay scales. U- Calculated based on the sum of ranks of the two groups being compared, Z- The standardized value of the U statistic, *p*-level of statistical significance.

Variable	U-Mann–Whitney Test for the Entire Cohort						
	Polyps Present (1)	Polyps Absent (0)	U	Z	N Polyps Present (1)	N Polyps Absent (0)	*p*
Zinreich CT scale before surgery	951.5	273.5	63.5	4.597	29	20	<0.001
Lund–Mackay CT scale before surgery	904.0	321.0	111.0	3.631	29	20	<0.001
Zinreich CT scale 24th week postoperatively	830.0	395.0	185.0	2.126	29	20	0.033
Lund–Mackay scale 24th week postoperatively	839.0	386.0	176.0	2.309	29	20	0.020

**Table 4:** Spearman’s rank coefficient results for the entire cohort and divided into subgroups regarding the tool used for the frontal beak reduction on the day of surgery and 7, 4 and 24 weeks after surgery). N-number, R-correlation strength [effect size], t(N-2)—the test statistic for significance testing, *p*-value—level of statistical significance.

SNOTT-22 Sum vs. Lund–Kennedy Score Sum	Spearman’s Rank Coefficient Entire Cohort				Spearman’s Rank Coefficient Piezo Knife				Spearman’s Rank Coefficient Drill			
	N	R	t(N-2)	*p*	N	R	t(N-2)	*p*	N	R	t(N-2)	*p*
On the day of surgery	49	0.135	0.937	0.354	25	0.354	1.817	0.082	24	0.056	0.263	0.795
1 week after surgery	49	0.198	1.388	0.172	25	0.179	0.873	0.392	24	0.271	1.322	0.200
4 weeks after surgery	49	0.172	1.197	0.237	25	0.244	1.208	0.239	24	0.104	0.493	0.627
24 weeks after surgery	49	0.110	0.756	0.454	25	0.064	0.307	0.762	24	0.193	0.920	0.367

## Data Availability

The full dataset supporting the findings is available from corresponding authors on request.
